# Long-Term Oncological Outcome Comparison between Intermediate- and High-Dose Radioactive Iodine Ablation in Patients with Differentiated Thyroid Carcinoma: A Propensity Score Matching Study

**DOI:** 10.1155/2021/6642971

**Published:** 2021-02-24

**Authors:** Kwangsoon Kim, Ja Seong Bae, Jeong Soo Kim

**Affiliations:** Department of Surgery, College of Medicine, The Catholic University of Korea, Seoul, Republic of Korea

## Abstract

**Background:**

Radioactive iodine (RAI) ablation is recommended for most patients with differentiated thyroid carcinoma (DTC) after total thyroidectomy (TT). We aimed to compare long-term outcomes between intermediate-dose (100 mCi) and high-dose (150 mCi) RAI ablation therapy in patients with DTC using propensity score matching analysis.

**Methods:**

This was a retrospective study of 1448 patients with DTC who underwent RAI ablation after TT. Propensity score matching was performed using the extent of operation, tumor size, extrathyroidal extension, multifocality, lymphatic invasion, vascular invasion, perineural invasion, number of positive lymph nodes (LNs), ATA risk stratification system, T stage, N stage, TNM stage, preoperative serum Tg and TgAb levels, and post-RAI serum Tg and TgAb levels.

**Results:**

Recurrence rates in the intermediate- and high-dose groups were 3.1% and 5.6%, respectively. After propensity score matching, LN ratio >0.22 (HR, 2.915; 95% CI, 1.228–6.918; *p*=0.015) and serum Tg >10 ng/mL after RAI (HR, 3.976; 95% CI, 1.839–8.595; *p* < 0.001) were significant predictors of recurrence. Kaplan–Meier analysis showed no significant difference in DFS before or after propensity score matching (*p*=0.074 and *p*=0.378, respectively**)**.

**Conclusions:**

Intermediate-dose RAI ablation for the adjuvant treatment of DTC is sufficient as compared to high-dose RAI ablation. Further prospective or multicenter studies should be conducted to clarify the prognosis of intermediate-dose RAI ablation.

## 1. Introduction

Differentiated thyroid carcinoma (DTC) is the most frequent endocrine malignancy. Its worldwide incidence has been increasing over the past several decades [1–3]. Papillary thyroid carcinoma (PTC) is the most common form of DTC and accounts for 90% of all thyroid malignancies, and the second most common form of DTC in Korea is follicular thyroid carcinoma (FTC) [4]. The development of diagnostic techniques and early screening has led to an increase in the diagnosis of papillary microcarcinoma [5, 6].

Management of DTC generally involves surgery followed by postoperative adjuvant therapy, including radioactive iodine (RAI) ablation and thyrotropin suppression [7]. The American Thyroid Association (ATA) management guidelines recommend a patient-individualized approach [8]. RAI ablation has been recommended for most DTC patients after total thyroidectomy (TT). RAI ablation has been recommended for most DTC patients after total thyroidectomy (TT). Its purpose is to eliminate normal remnant thyroid tissue to achieve undetectable serum thyroglobulin (Tg) and to eradicate any foci of carcinoma to prevent recurrence and to perform a diagnostic whole-body scanning to detect persistent thyroid carcinoma [8]. More recently, RAI ablation is being reserved for selected patients with higher recurrence risk after surgery alone. Although RAI ablation is usually safe, well tolerated, and effective in these patients, the optimal dose to maximize treatment effect while minimizing side effects remains controversial.

The side effects include transient neck pain, nausea, vomiting, loss of taste, acute and/or chronic salivary gland dysfunction, sialadenitis, temporary gonadal dysfunction, and rarely radiation-related second malignancy [9–11]. Since most side effects are dose-dependent, it is important to use the minimal RAI dose that will achieve the maximal ablation effect for the patient's quality of life [12].

Many previous studies comparing oncologic outcomes after various RAI doses in DTC patients have reported different outcomes [13–17]. To the best of our knowledge, this is one of the largest case series to date comparing the long-term oncological outcomes between intermediate- and high-dose RAI ablation. Therefore, the objective of this study was to compare long-term outcomes between intermediate- and high-dose RAI ablation therapy in patients with DTC using propensity score matching analysis in a large series of patients.

## 2. Materials and Methods

### 2.1. Patients

We retrospectively reviewed the medical records of 1539 patients with DTC who underwent RAI ablation after TT and/or modified radical neck dissection (mRND) from January 2009 to December 2014 at Seoul St. Mary's Hospital (Seoul, Korea). After excluding 18 and 73 patients due to inadequate data and loss of follow-up, respectively, 1448 patients were analyzed. Patients were categorized into two groups according to RAI dose, intermediate dose (100 mCi), and high dose (150 mCi). The lymph node (LN) ratio was defined as the number of positive LNs divided by the number of harvested LNs. The mean follow-up duration was 92.2 ± 23.4 months (range, 62–134). This study was conducted in accordance with the Declaration of Helsinki (as revised in 2013). This study was approved by the institutional review board of Seoul St. Mary's Hospital, The Catholic University of Korea (IRB No : KC20RISI0278), which waived the requirement for informed consent due to the retrospective nature of this study.

### 2.2. Postoperative Management and Follow-Up

All patients were managed according to ATA management guidelines [8] after surgical treatment and received suppressive doses of levothyroxine. Regular follow-up consisted of physical examination, thyroid function and antithyroglobulin antibody level testing, and ultrasonography of the neck every 3–6 months and annually thereafter. Additional diagnostic imaging modalities, such as computed tomography and positron emission tomography/computed tomography, were performed as necessary to confirm the recurrent disease.

### 2.3. RAI Protocol

RAI ablation was performed 6–8 weeks after surgery by an experienced nuclear medicine physician. All study patients underwent thyroid hormone withdrawal for at least 4 weeks prior to RAI ablation or two daily injections of 0.9 mg recombinant human thyroid-stimulating hormone (TSH) for TSH stimulation. Patients have also prescribed a low iodine diet before ablation. RAI was administered once the serum TSH level was >30 mUI/L to enhance iodine uptake into the remnant tissue and allow the delivery of a higher radiation dose. The initial RAI dose was 100 mCi (intermediate-dose) in 1146 patients and 150 mCi (high-dose) in 302 patients. Radioiodine whole-body scintigraphy and single-proton emission computed tomography were performed 5–7 days after RAI ablation.

### 2.4. Laboratory Studies

Patients underwent venipuncture to collect a blood sample for measurement of serum Tg and serum Tg antibody (TgAb) levels. Serum Tg was measured using an immunoradiometric Tg assay (CIS Bio International, Saclay, France) with a functional sensitivity of 0.2 ng/mL. Serum TgAb concentration was measured using a radioimmunoassay (DIAsource, Rue du Bosquet, Belgium); serum TgAb level <60 IU/mL was considered negative [18]. The Tg ratio was defined as post-RAI serum Tg level/preoperative serum Tg level.

### 2.5. Statistical Analysis

Continuous and quantitative data are reported as means with standard deviation, and categorical qualitative data are presented as numbers with percentages. The student's *t*-test was used to compare continuous variables; categorical variables were compared using Pearson's chi-square test or Fisher's exact test. Univariate and multivariate Cox regression analyses were performed to identify disease-free survival (DFS) predictors using calculated hazard ratios (HRs) with 95% confidence intervals (CIs). Kaplan–Meier DFS curves were compared using the log-rank test. Receiver operating characteristics (ROC) curve analysis was performed to determine the optimal cutoff values for the LN ratio and Tg ratio.

To reduce the impact of selection bias and potential confounding factors, propensity score matching was performed using sixteen clinicopathological and biochemical characteristics: extent of operation, tumor size, extrathyroidal extension, multifocality, lymphatic invasion, vascular invasion, perineural invasion, number of positive LNs, ATA risk stratification system, T stage, N stage, TNM stage, preoperative serum Tg and TgAb levels, and post-RAI serum Tg and TgAb levels. Individual patient propensity scores were calculated using logistic regression analysis. After propensity score matching, the baseline clinicopathological and biochemical characteristics representative of long-term oncologic outcomes were compared between the two groups. DFS after propensity score matching was compared using Kaplan–Meier survival analysis with the log-rank test in the same way as before propensity score matching. *p* < 0.05 was considered significant. All statistical analyses were performed using SPSS software version 24.0 for Windows (IBM Corp., Armonk, NY, USA).

## 3. Results

### 3.1. Comparison of Baseline Clinicopathological Characteristics between the Intermediate- and High-Dose Groups before and after Propensity Score Matching

The baseline clinicopathological characteristics of the intermediate- and high-dose groups are shown in Table 1. Mean age, gender, type of carcinoma, bilaterality, lymphatic invasion, vascular invasion, BRAF^V600E^ positivity, and the number of harvested LNs did not significantly differ between groups. A significantly greater proportion of patients in the high-dose group underwent more extensive surgery (TT and mRND) compared to the intermediate-dose group (24.5% vs. 16.8%, *p*=0.003). Tumor size was significantly larger in the high-dose group (1.4 ± 0.9 cm vs. 1.2 ± 0.8 cm, *p*=0.001). The high-dose group had a significantly higher prevalence of extrathyroidal extension (ETE) but a significantly lower prevalence of multifocality (19.2% vs. 8.1%, *p* < 0.001; and 44.0% vs. 53.8%, *p*=0.003, respectively). Although the number of harvested LNs did not significantly differ between the groups, the number of positive LNs was significantly greater in the high-dose group (5.2 ± 6.0 vs. 4.0 ± 5.9 cm, *p*=0.004). In terms of ATA risk stratification, the high-dose group had a significantly higher risk of recurrence (*p* < 0.001). Patients in the high-dose group were diagnosed with significantly higher T stage, N stage, and TNM stage tumors (*p* < 0.001, *p* < 0.001, and *p*=0.008, respectively). Thirty-five (3.1%) patients in the intermediate-dose group and 17 (5.6%) patients in the high-dose group experienced recurrence; the difference was significant (*p*=0.038).

Propensity score matching yielded 552 matched patient pairs. There were no differences in baseline clinicopathological characteristics between the matched groups (Table 1).

### 3.2. Comparison of Perioperative Biochemical Characteristics between the Intermediate- and High-Dose Groups before and after Propensity Score Matching

Table 2 shows the perioperative biochemical characteristics of the intermediate- and high-dose groups. Preoperative TSH level, preoperative serum Tg level, the ratio of preoperative serum Tg of 1 ng/mL, the ratio of positivity of preoperative serum TgAb, and the ratio of positivity of serum TgAb after RAI did not significantly differ between the intermediate- and high-dose groups. However, preoperative serum Tg >10 ng/mL was significantly higher in the high-dose group (63.9% vs. 55.9%, *p*=0.013). The high-dose group had a significantly higher ratio of serum Tg >1 ng/mL and >10 ng/mL after RAI (49.0% vs. 42.0%, *p*=0.031; and 18.5% vs. 8.6%, *p* < 0.001, respectively). After propensity score matching, there were no differences in perioperative biochemical characteristics between the groups (Table 2).

### 3.3. Univariate and Multivariate Analyses of Recurrence Risk Factors before and after Propensity Score Matching

Table 3 presents the results of univariate and multivariate Cox regression analyses before propensity score matching. Perineural invasion (HR, 2.918; 95% CI, 1.147–5.217; *p*=0.025), number of positive LNs (HR, 1.039; 95% CI, 0.109–1.059; *p* < 0.001), LN ratio >0.22 (HR, 2.373; 95% CI, 1.260–4.469; *p*=0.007), and positive serum TgAb after RAI (HR, 2.564; 95% CI, 1.197–5.508; *p*=0.015) were significant predictors of recurrence. The most significant predictor of recurrence was serum Tg >10 ng/mL after RAI (HR, 4.504; 95% CI, 2.521–8.045; *p* < 0.001). After propensity score matching, LN ratio >0.22 (HR, 2.915; 95% CI, 1.228–6.918; *p*=0.015) and serum Tg >10 ng/mL after RAI (HR, 3.976; 95% CI, 1.839–8.595; *p* < 0.001) were confirmed as significant predictors of recurrence (Table 4).

**Figure 1:**
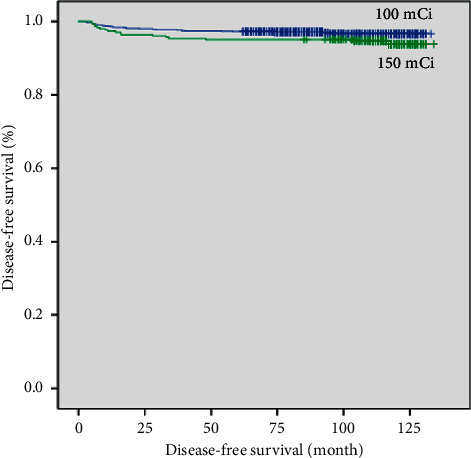
Disease-free survival curves of intermediate-dose (100 mCi) and high-dose (150 mCi) groups before propensity score matching (*p*=0.074).

**Figure 2:**
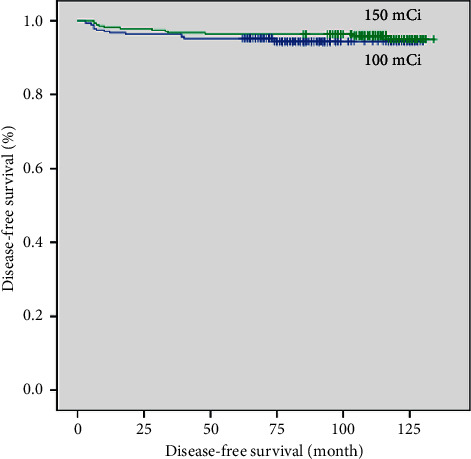
Disease-free survival curves of intermediate-dose (100 mCi) and high-dose (150 mCi) groups after propensity score matching (*p*=0.378).

**Figure 3:**
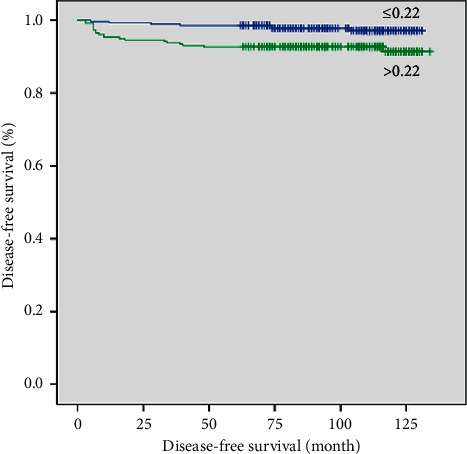
Disease-free survival curves according to post-RAI serum Tg level after propensity score matching (cut-off, 10 ng/mL; *p* < 0.001).

**Figure 4:**
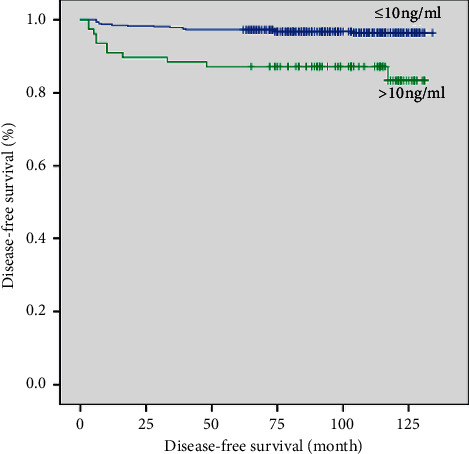
Disease-free survival curves according to lymph node ratio after propensity score matching (cut-off, 0.22; *p*=0.005).

In the Kaplan–Meier analysis, DFS did not significantly differ between the intermediate- and high-dose groups before or after propensity score matching (*p*=0.074 and *p*=0.378, respectively; Figures 1 and 2). After propensity score matching, multivariate Cox regression analysis identified LN ratio >0.22 and serum Tg >10 ng/mL after RAI as significant predictors of recurrence. DFS according to LN ratio and serum Tg after RAI was significantly different between the two groups as well (*p*=0.005 and *p* < 0.001, respectively; Figures 3 and 4).

### 3.4. Subgroup Analysis of the Intermediate- and High-Dose Groups according to ATA Risk Stratification after Propensity Score Matching

For subgroup outcome analysis according to ATA risk stratification, the patients were divided into intermediate-risk (*n* = 390) and high-risk (*n* = 84) groups. In the intermediate-risk group, there was no significant difference in recurrence between the intermediate- and high-dose patients (6.0% vs 5.9%, *p*=0.979). In the high-risk group, there was no significant difference in recurrence according to dose as well (4.4% vs 2.6%, *p*=0.643) (Table 5).

## 4. Discussion

The management of thyroid carcinoma is individualized and must take into account risk factors for death and recurrence. After RAI was first proposed for therapeutic use in patients with hyperthyroidism by researchers at the Massachusetts General Hospital [19], it has been widely used in patients with DTC and is considered a first-line adjuvant therapy after TT [8].

RAI ablation is not routinely recommended after TT for patients with unifocal and intrathyroidal papillary microcarcinoma without other high-risk factors [20, 21]. However, RAI ablation should be considered in ATA intermediate-risk patients [22] and is routinely recommended for those with high risk [8, 23]. Thus, postoperative ATA risk classification plays an important role in determining the use of RAI ablation. Additional considerations may include patient comorbidities, patient preferences, and preferred disease surveillance procedures [14].

Numerous studies have examined the effect of RAI ablation on recurrence and mortality in patients with DTC. Mazzaferri et al. reported that RAI ablation significantly reduced both recurrence and mortality [24], and Sawka et al. reported that RAI ablation significantly reduced recurrence and distant metastasis. As a result of these studies, RAI ablation became the standard treatment for patients with DTC after TT [25]. However, Jonklaas et al. found no survival benefit after RAI ablation in TNM stage I DTC patients [26], and Schvartz et al. found that RAI ablation in ATA low-risk DTC patients had no impact on recurrence or mortality [27]. Thus, RAI ablation remains controversial in ATA low-risk DTC patients. In 2015, the ATA recommended using RAI ablation in DTC patients according to recurrence risk [8].

This study compared long-term outcomes between intermediate- and high-dose RAI ablation using recurrence and DFS as measures. The high-dose group was significantly associated with more aggressive tumor characteristics, including larger tumor size, higher prevalence of ETE, higher number of positive LNs, higher ATA risk classification, higher TNM stage, and higher preoperative and postablation serum Tg level. Therefore, confounding and selection bias may have been introduced. To minimize their effects, propensity score matching analysis was performed to adjust for several clinicopathological characteristics.

Although the recurrence rate was significantly higher in the high-dose group before propensity score matching (3.1% vs. 5.6%, *p*=0.038), after matching, the recurrence rates were similar (5.4% vs. 4.3%, *p*=0.694), and there was no significant difference in DFS (log-rank *p*=0.378). Only LN ratio >0.22 (HR, 2.915, *p*=0.015) and serum Tg level >10 ng/mL after RAI (HR, 3.976, *p* < 0.001) were identified as significant recurrence risk factors in the multivariate analysis, not RAI dose. Several studies have reported that high-dose RAI ablation has no major advantage over low-dose [13, 28, 29]. A meta-analysis by Cheng et al. validated that low-dose RAI was sufficient for thyroid remnant ablation as compared to high-dose with similar quality of life, less common side effects, and a shorter hospital stay [30]. However, to the best of our knowledge, this is the first study to compare long-term oncologic outcomes between intermediate- and high-dose RAI ablation using propensity score matching analysis.

Sacks et al. reported that low-risk patients achieve no survival or DFS benefit from RAI ablation [31]. This study included patients with intermediate- and high-risk ATA classifications in the subgroup analysis and confirmed that RAI dose did not affect recurrence in either (6.0% vs 5.9%, *p*=0.979; 4.4% vs 2.6%, *p*=0.643, respectively).

RAI ablation-related side effects can be divided into those related to ablation preparation and those related to radiation. In preparation for effective RAI ablation, levothyroxine should be discontinued to increase serum TSH. A hypothyroidism state may then result and manifest with various symptoms, such as weight gain, fatigue, cold intolerance, hypothermia, muscle cramps, and constipation. Alternatively, in low- or intermediate-risk patients, recombinant human TSH can be administered instead of discontinuing levothyroxine [8, 32]. Although RAI ablation is safe, radiation-related side effects are dose-dependent [12] and can occur even at relatively low doses [6, 33]. These include salivary gland dysfunction, temporary gonadal dysfunction, and secondary malignancy [34–36]. Higher RAI doses can lower the patient's quality of life without providing treatment benefits. This study suggests that a lower RAI dose can achieve an equivalent treatment effect with fewer side effects.

This study has several limitations. Its retrospective single-center design may have introduced selection bias. However, propensity score matching was performed to adjust for differences in clinicopathological characteristics and minimize bias. In addition, we included patients of all ATA risk classifications, from low to high risk. Although we performed a subgroup analysis of intermediate- and high-risk patients, this may be a limitation. Moreover, we did not evaluate side effects related to RAI ablation, which are dose-dependent. Although an examination of side effects would have been beneficial, their objective evaluation is difficult as most side effects are subjective in nature. Finally, the follow-up period of this study was relatively short, which limited the ability to compare long-term oncological outcomes between the intermediate- and high-dose groups. Future studies evaluating prognostic factors in patients with DTC will require longer follow-up because of the indolent nature of the disease. Nonetheless, this study's strength is its follow-up of every patient and use of a standardized laboratory and imaging protocol in a single institution.

## 5. Conclusions

Intermediate-dose RAI ablation for the adjuvant treatment of DTC is sufficient as compared to high-dose RAI ablation. To the best of our knowledge, this is the first study to compare the long-term oncological outcome between intermediate- and high-dose RAI ablation. Further prospective or multicenter studies are warranted to clarify the prognosis of intermediate-dose RAI ablation.

## Figures and Tables

**Table 1:** Comparison of baseline clinicopathological characteristics between intermediate- (100 mCi) and high-dose (150 mCi) groups before and after propensity score matching.

	Before matching		*p* value	After matching		*p* value
	100 mCi (*n* = 1146)	150 mCi (*n* = 302)		100 mCi (*n* = 276)	150 mCi (*n* = 276)	
Age (years)	46.3 ± 12.2 (range, 11–81)	45.9 ± 12.7 (range, 18–74)	0.599	45.6 ± 13.3 (range, 12–81)	45.6 ± 12.8 (range, 18–74)	0.997
≥55	294 (25.7%)	84 (27.8%)	0.462	201 (72.8%)	202 (73.2%)	0.924
<55	852 (74.3%)	218 (72.2%)		75 (27.2%)	74 (26.8%)	

Female	869 (75.8%)	238 (78.8%)	0.287	206 (74.6%)	214 (77.5%)	0.485
Type of carcinoma			0.185			1.000
PTC	1137 (99.2%)	297 (98.3%)		272 (98.6%)	272 (98.6%)	
FTC	9 (0.8%)	5 (1.7%)		4 (1.4%)	4 (1.4%)	

Extent of operation			0.003			0.616
TT	953 (83.2%)	228 (75.5%)		208 (75.4%)	214 (77.5%)	
TT and mRND	193 (16.8%)	74 (24.5%)		68 (24.6%)	62 (22.5%)	

Tumor size (cm)	1.2 ± 0.8 (range, 0.2–9.0)	1.4 ± 0.9 (range, 0.2–6.5)	0.001	1.4 ± 0.8 (range, 0.2–5.4)	1.3 ± 0.8 (range, 0.2–6.5)	0.513
ETE	93 (8.1%)	58 (19.2%)	<0.001	48 (17.4%)	40 (14.5%)	0.416
Multifocality	617 (53.8%)	133 (44.0%)	0.003	133 (48.2%)	127 (46.0%)	0.670
Bilaterality	416 (36.3%)	99 (32.8%)	0.280	85 (30.8%)	94 (34.1%)	0.467
Lymphatic invasion	491 (42.8%)	147 (48.7%)	0.078	139 (50.4%)	128 (46.4%)	0.394
Vascular invasion	38 (3.3%)	17 (5.6%)	0.088	15 (5.4%)	15 (5.4%)	1.000
Perineural invasion	28 (2.4%)	19 (6.3%)	0.002	12 (4.3%)	10 (3.6%)	0.828
BRAF^V600E^ positive	700/880 (79.5%)	126/158 (79.7%)	0.954	173/216 (80.1%)	111/140 (79.3%)	0.893
Harvested LNs	19.6 ± 21.6	20.4 ± 21.7	0.554	22.9 ± 23.4	19.5 ± 21.3	0.073
Positive LNs	4.0 ± 5.9	5.2 ± 6.0	0.004	4.9 ± 5.7	4.9 ± 5.9	0.901

ATA risk stratification			<0.001			0.740
Low	273 (23.8%)	48 (15.9%)		47 (17.0%)	51 (18.5%)	
Intermediate	787 (68.7%)	197 (65.2%)		184 (66.7%)	186 (67.4%)	
High	86 (7.5%)	57 (18.9%)		45 (16.3%)	39 (14.1%)	

T stage			<0.001			0.773
T1	953 (83.2%)	215 (71.2%)		200 (72.5%)	208 (75.4%)	
T2	85 (7.4%)	27 (8.9%)		27 (9.8%)	26 (9.4%)	
T3a	15 (1.3%)	2 (0.7%)		1 (0.4%)	2 (0.7%)	
T3b	90 (7.9%)	55 (18.2%)		47 (17.0%)	38 (13.8%)	
T4	3 (0.3%)	3 (1.0%)		1 (0.4%)	2 (0.7%)	

N stage			<0.001			0.820
N0	284 (24.8%)	52 (17.2%)		47 (17.0%)	50 (18.1%)	
N1a	669 (58.4%)	176 (58.3%)		161 (58.3%)	164 (59.4%)	
N1b	193 (16.8%)	74 (24.5%)		68 (24.6%)	62 (22.5%)	

TNM stage			0.008			0.608
Stage I	939 (81.9%)	228 (75.5%)		218 (79.0%)	212 (76.8%)	0.694
Stage II	207 (18.1%)	73 (24.2%)		58 (21.0%)	64 (23.2%)	
Stage III	0 (0%)	1 (0.3%)		0 (0%)	0 (0%)	

Recurrence	35 (3.1%)	17 (5.6%)	0.038	15 (5.4%)	12 (4.3%)

Data are expressed as the patient's number (%) or mean ± SD. A statistically significant difference was defined as *p* < 0.05. Abbreviation: PTC, papillary thyroid carcinoma; FTC, follicular thyroid carcinoma; TT, total thyroidectomy; mRND, modified radical neck dissection; ETE, extrathyroidal extension; LN, lymph node; ATA, American thyroid association; T, tumor; N, node; M, metastasis.

**Table 2:** Comparison of perioperative biochemical characteristics between intermediate- (100 mCi) and high-dose (150 mCi) groups before and after propensity score matching.

	Before matching		*p* value	After matching		*p* value
	100 mCi (*n* = 1146)	150 mCi (*n* = 302)		100 mCi (*n* = 1146)	150 mCi (*n* = 302)	
Pre-op. TSH (mIU/L)	2.9 ± 10.9	2.2 ± 1.8	0.276	2.1 ± 1.5	2.2 ± 1.8	0.549
Pre-op. serum Tg (ng/mL)	30.9 ± 75.6	39.7 ± 89.4	0.086	38.7 ± 99.9	39.9 ± 92.4	0.885
≤1	114 (9.9%)	30 (9.9%)	1.000	30 (10.9%)	28 (10.1%)	0.890
>1	1032 (90.1%)	272 (90.1%)		246 (89.1%)	248 (89.9%)	
≤10	505 (44.1%)	109 (36.1%)	0.013	111 (40.2%)	99 (35.9%)	0.335
>10	641 (55.9%)	193 (63.9%)		165 (59.8%)	177 (64.1%)	

Pre-op. serum TgAb (IU/mL)	113.7 ± 595.9	207.0 ± 884.2	0.031	170.5 ± 889.8	144.3 ± 569.0	0.681
Negative	922 (80.5%)	235 (77.8%)	0.333	225 (81.5%)	216 (78.3%)	0.396
Positive	224 (19.5%)	67 (22.2%)		51 (18.5%)	60 (21.7%)	

Post-RAI serum Tg (ng/mL)	3.8 ± 12.5	9.8 ± 41.2	<0.001	5.3 ± 13.7	6.7 ± 15.9	0.263
≤1	665 (58.0%)	154 (51.0%)	0.031	141 (51.1%)	144 (52.2%)	0.865
>1	481 (42.0%)	148 (49.0%)		135 (48.9%)	132 (47.8%)	
≤10	1048 (91.4%)	246 (81.5%)	<0.001	245 (88.8%)	229 (83.0%)	0.066
>10	98 (8.6%)	56 (18.5%)		31 (11.2%)	47 (17.0%)	

Post-RAI serum TgAb (IU/mL)	31.1 ± 100.9	64.2 ± 324.8	0.003	44.5 ± 163.9	35.6 ± 133.3	0.484
Negative	1067 (93.1%)	272 (90.1%)	0.086	252 (91.3%)	252 (91.3%)	1.000
Positive	79 (6.9%)	31 (9.9%)		24 (8.7%)	241 (8.7%)	

Data are expressed as the patient's number (%) or mean ± SD. A statistically significant difference was defined as *p* < 0.05. Negative TgAb, TgAb <60 IU/mL; positive TgAb, TgAb ≥60 IU/mL. Abbreviation: TSH, thyroid-stimulating hormone; pre-op, preoperative; post-RAI, after radioactive iodine therapy; Tg, thyroglobulin; TgAb, thyroglobulin antibody.

**Table 3:** Univariate and multivariate analyses of recurrence risk factors before propensity score matching.

	Univariate		Multivariate	
	HR (95% CI)	*p* value	HR (95% CI)	*p* value
Age	0.962 (0.940–0.984)	0.001		
Extent of operation				
TT	Ref.			
TT and mRND	2.009 (1.114–3.621)	0.020		
Tumor size	1.382 (1.119–1.707)	0.003		
ETE	2.629 (1.379–5.013)	0.003		
Lymphatic invasion	2.734 (1.531–4.884)	0.001		
Vascular invasion	4.114 (1.855–9.123)	0.001		
Perineural invasion	3.185 (1.267–8.010)	0.014	2.918 (1.147–5.217)	0.025
Harvested LNs	1.017 (1.008–1.025)	<0.001		
Positive LNs	1.062 (1.045–1.080)	<0.001	1.039 (1.019–1.059)	<0.001
LN ratio				
≤0.22	Ref.		Ref.	
>0.22	3.513 (1.928–6.401)	<0.001	2.373 (1.260–4.469)	0.007
ATA risk stratification				
Low	Ref.			
Intermediate	4.348 (1.344–14.069)	0.014		
High	7.734 (2.128–28.104)	0.002		
T stage				
T3b	2.919 (1.511–5.637)	0.001		
N stage				
N0	Ref.			
N1a	2.589 (1.006–6.662)	0.048		
N1b	4.264 (1.561–11.650)	0.005		
Pre-op. serum Tg	1.003 (1.002–1.005)	<0.001		
≤10 ng/mL	Ref.			
>10 ng/mL	2.242 (1.197–4.201)	0.012		
Post-RAI serum Tg	1.005 (1.002–1.008)	0.001		
≤1 ng/mL	Ref.			
>1 ng/mL	3.257 (1.788–5.934)	<0.001		
≤10 ng/mL	Ref.		Ref.	
>10 ng/mL	5.976 (3.434–10.401)	<0.001	4.504 (2.521–8.045)	<0.001
Post-RAI serum TgAb				
Negative	Ref.		Ref.	
Positive	2.271 (1.069–4.825)	0.033	2.567 (1.197–5.508)	0.015
Tg. ratio				
≤0.077	Ref.			
>0.077	2.264 (1.256–4.079)	0.007		

Data are expressed as hazard ratio (HR) and 95% confidence interval (CI). A *p* value <0.05 was considered statistically significant. LN ratio is defined as the number of positive LNs divided by the number of harvested LNs. Tg ratio is defined as post-RAI Tg/preop Tg. Abbreviation: TT, total thyroidectomy; mRND, modified radical neck dissection; ETE, extrathyroidal extension; LN, lymph node; T, tumor; N, node; ATA, American thyroid association; pre-op, preoperative; post-RAI, after radioactive iodine therapy; Tg, thyroglobulin; TgAb, thyroglobulin antibody.

**Table 4:** Univariate and multivariate analyses of recurrence risk factors after propensity score matching.

	Univariate		Multivariate	
	HR (95% CI)	*p* value	HR (95% CI)	*p* value
Age	0.960 (0.931–0.990)	0.009		
Lymphatic invasion	2.645 (1.158–6.043)	0.021		
Positive LNs	1.068 (1.024–1.114)	0.002		
LN ratio				
≤0.22	Ref.		Ref.	
>0.22	3.222 (1.362–7.622)	0.008	2.915 (1.228–6.918)	0.015
Pre-op. serum Tg	1.002 (1.000–1.004)	0.046		
≤10 ng/mL	Ref.			
>10 ng/mL	3.652 (1.263–10.560)	0.017		
Post-RAI serum Tg	1.018 (1.005–1.031)	0.006		
≤1 ng/mL	Ref.			
>1 ng/mL	3.125 (1.321–7.391)	0.009		
≤10 ng/mL	Ref.		Ref.	
>10 ng/mL	4.385 (2.034–9.451)	<0.001	3.976 (1.839–8.595)	<0.001

Data are expressed as hazard ratio (HR) and 95% confidence interval (CI). A *p* value <0.05 was considered statistically significant. LN ratio is defined as the number of positive LNs/the number of harvested LNs. Tg ratio is defined as post-RAI Tg/preop Tg. Abbreviation: TT, total thyroidectomy; mRND, modified radical neck dissection; ETE, extrathyroidal extension; LN, lymph node; T, tumor; N, node; ATA, American thyroid association; pre-op, preoperative; post-RAI, after radioactive iodine therapy; Tg, thyroglobulin; TgAb, thyroglobulin antibody.

**Table 5:** Subgroup analysis between intermediate- (100 mCi) and high-dose (150 mCi) groups according to the ATA risk stratification after propensity score matching.

*Intermediate-risk*	*100 mCi (n = 184)*	*150 mCi (n = 186)*	*pvalue*
Age (years)	45.4 ± 14.1 (range, 20–65)	45.3 ± 13.3 (range, 16–80)	0.986
Female	135 (73.4%)	141 (75.8%)	0.643
Tumor size (cm)	1.4 ± 0.9	1.4 ± 0.9	0.739
T stage			0.840
T1/T2/T3a/T3b/T4	157 (85.2%)/23 (12.5%)/1 (0.6%)/2 (1.1%)/1 (0.6%)	162 (87.1%)/22 (11.8%)/1 (0.5%)/1 (0.5%)/0	
N stage			0.577
N0/N1a/N1b	37 (20.1%)/101 (54.9%)/46 (25.0%)	32 (17.2%)/112 (60.2%)/42 (22.6%)	
Pre-op. serum Tg (ng/mL)	44.3 ± 113.1	41.2 ± 96.6	0.774
Post-RAI serum Tg (ng/mL)	5.3 ± 14.5	7.7 ± 17.3	0.137
Recurrence	11 (6.0%)	11 (5.9%)	0.979

*High-risk*	*100 mCi (n = 45)*	*150 mCi (n = 39)*	*pvalue*
Age (years)	47.6 ± 11.7 (range, 12–78)	50.2 ± 12.4 (range, 14–84)	0.338
Female	37 (72.9%)	37 (78.5%)	0.097
Tumor size (cm)	1.6 ± 0.8	1.4 ± 0.6	0.068
T stage			0.213
T1/T2/T3a/T3b/T4	0/0/0/45 (100%)/0	0/0/0/37 (94.9%)/2 (5.1%)	
N stage			0.114
N0/N1a/N1b	4 (8.9%)/23 (51.1%)/18 (40%)	10 (25.6%)/15 (38.5%)/14 (35.9%)	
Pre-op. serum Tg (ng/mL)	23.6 ± 31.0	53.3 ± 118.7	0.109
Post-RAI serum Tg (ng/mL)	4.3 ± 10.0	3.4 ± 5.9	0.612
Recurrence	2 (4.4%)	1 (2.6%)	0.643

Data are expressed as the patient's number (%) or mean ± SD. A statistically significant difference was defined as *p* < 0.05. Abbreviation: ATA, American thyroid association; T, tumor; N, node; pre-op, preoperative; post-RAI, after radioactive iodine therapy; Tg, thyroglobulin.

## Data Availability

The data that support the findings of this study are available on request from the corresponding author. The data are not publicly available due to privacy or ethical restrictions.
